# Posttraumatic proliferating trichilemmal tumour on the frontal region of the scalp: a case report

**DOI:** 10.1186/1757-1626-3-80

**Published:** 2010-03-20

**Authors:** Ilker Sengul, Demet Sengul

**Affiliations:** 1 Department of General Surgery, Giresun University Faculty of Medicine, 28100 Giresun/Turkey; 2 Department of Pathology, Prof. Dr. A. Ilhan Ozdemir State Hospital, 28100 Giresun/Turkey

## Abstract

**Introduction:**

Proliferating trichilemmal tumour defined with more than one terms by many author, after well documentated series reported as "proliferating epidermoid cysts" by Wilson-Jones, firstly in 1966. They are rare, slowly growing, lobular masses inherited autosomal dominantly and localized on scalps of older women and believed to arising as a complication of a trauma and inflammation and effect 5-10% of people.

**Case presentation:**

We intented to present the case of a 62 years old Turkish woman with a history of slowly growing scalp mass after the trauma, especially during last 15 years. After surgical evaluation, histopathological slides exhibited the characteristic structures of proliferating trichilemmal tumour. The patient was lost to follow-up and no recurrens or distance metastasis detected during 40 months follow-up.

**Conclusion:**

In our opinion, widely surgical excision with long-term surveillance is the best choice for both diagnosis and treatment still today.

## Introduction

Besides terms of proliferating trichilemmal cyst (PTC) and proliferating trichilemmal tumour (PTT), it is called as the terms like invasive pilomatrixoma, trichochlamydocarcinoma, giant hair matrix tumour, scalp pilar tumour, trichilemmal pilar tumour, proliferating follicular cystic neoplasm [1] by many person, after well documentated series reported as "proliferating epidermoid cysts" by Wilson-Jones, firstly in 1966 [2]. PTCs, also known as pilar tumours are slowly growing, lobular masses and localized on scalps of older women and believed to arising as a complication of a trauma and inflammation. While trichilemmal cysts, which is inherited autosomal dominantly effect 5 - 10% of people, they are seen more frequently in women older than 50 years [3].

## Case presentation

62 years old Turkish woman having the history of trauma to her 0.5 × 0.5 cm mass on her frontal region of scalp 46 years ago, applied to our clinic. There was a history of growing slowly and complaint of itch on the described mass after the trauma especially during last 15 years.

On physical examination, there was a mass lined with hair on the frontal region, 4 cm superior to starting point of hair of forehead and partially ulcerated, fluctuated, size of which is approximately 2.5 × 2 cm. There was no fixation to underlying area of bone and no lymph nodes were palpabl in the neck. The clinical diagnosis was made as sebaceous cyst and the lesion was totally excised.

**Figure 1:**
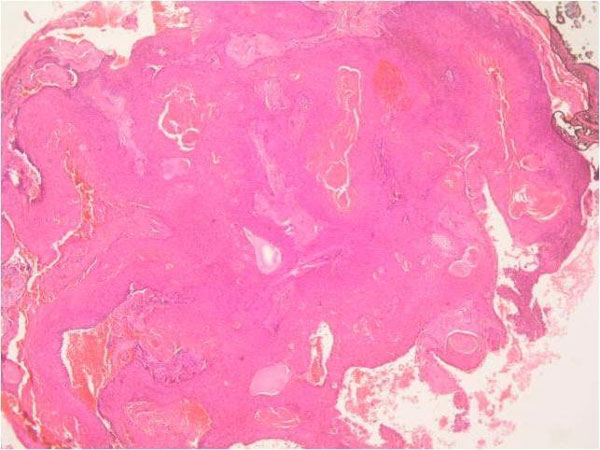
**Trichilemmal type keratinization and focal epidermal keratinization, mitosis, moderate mononuclear inflammatory infiltrate areas, eosinophilic center surrounded with stratified squamous epithelium and lobulated cyst wall filled with squamous epithelium of the proliferating trichilemmal tumour (Haematoxylin & Eosin, Original magnification ×50)**.

The resected tissue was set in 10% formalin for histopathological examination. Macroscopically, the tissue was rubbery firm in consistency, filled with tan brown necrotic material covered with hairy skin and contain ulcerated skin material on it which constitude nearly 2/3 of surface of the whole mass. Microscopically, histopathological slides revealed the characteristic structures of PTT. Trichilemmal type keratinization and focal epidermal keratinization, bands of squamous epithelium and within the bands basaloid cells at periphery, hemorrhagic areas within the cystic cavity, mitosis, moderate mononuclear inflammatory infiltrate areas, eosinophilic center surrounded with stratified squamous epithelium and lobulated cyst wall filled with squamous epithelium were observed (Figure 1). According to microscopic examination with classical haematoxylin & eosin (H & E), the diagnosis of PTT was made finally. The patient was lost to follow-up and no recurrens or distance metastasis were detected during 40 months follow-up.

## Discussion

PTTs are the masses localized in dermis or subcutaneous tissue, may become exophytic, sometimes exhibits ulceration, and are solid or partially cystic. The size of which ranges from 2 to 15 cm [4]; but also a giant hair matrix tumour is sized 25 cm in largest dimension was reported in the literature [5]. Ordinarily, the lesion is encountered on the scalp; but wrist, elbow, mons pubis, vulva, buttock, and chest are other locations where it can be found. Duration of lesion ranges between 4 to 50 years in the literature [6].

Histologically, PTT exhibit tichilemmal keratinization areas. However, some cases may include the areas of focal epidermal keratinization. They comprise eosinophilic center is surrounded with stratified squamous epithelium and lobulated cyst wall is filled with squamous epithelium (Figure 1).

As the oncological transformation of trichilemmal tumour, Saida et al. [7] defined the three stages: trichilemmal cyst, the adenomatous stage; proliferating trichilemmal cyst, the epitheliomatous stage; malignant PTT, the carcinomatous stage. Nevertheless, malignant transformation to squamous cell carcinoma or spindle cell (sarcomatoid) carcinoma is a rare phenomenon [8]. Microscopically, the charecteristics of benign PTCs seem like the charecteristic of squamous cell carcinoma. Pilar tumours always include foci of squamous differentiation resembling squamous cell carcinoma [3]. However, thought of PTT may be a variant of squamous cell carcinoma is not broadly aquiescenced [9]. While trichilemmal cyst formation, trichilemmal type keratinization, eosinophilic hyaline membrane, calcification, absence of premalignant epidermal lesion such as Bowen's disease or actinic keratosis are features favoring the diagnosis of PTT [3]; extensive cellular atypia and invasion of adjacent structures are usually necessary for diagnosis of malignant PTT [4].

Contrary to simple trichilemmal cysts, widely local excision, additional reconstruction if necessary and long term follow-up is required in these tumours to prevent recurrence [3]. It is also necessary for PTCs because of their malignant potential [10]. In the differantial diagnosis, Brooke-Spiegler syndrome, cylindroma, dermoid cyst, squamous cell carcinoma [8]. Brooke-Spiegler syndrome exhibits variable expression and penetrance presenting with multiple cylindromas and trichoepitheliomas and is a rare autosomal dominant disorder [10], appearing on the head and neck region [11]. It results from mutations or loss of heterozygosity of the cylindromatosis gene (CYLD) located at 16q12-q13 [[20], 21, 22] is a tumour suppressor gene. Although it is seldom, malignant transformation to cylindrocarcinomas and metastasis can occur in cylindroma [12]; likely malignant transformation to BCC can set in trichoepitheliomas [13]. Excision, dermabrasion, electrodessication, CO_2 _laser, cryotherapy, radiotherapy or topical applications of aspirin derivatives are reported treatment modalities of cylindromas [14]. For the unwelcome recurrence rates and the risk of malignant transformation of these tumors, wide local excision is the favored method of treatment [15].

Squamous cell carcinoma is the second leading cause of skin cancer in whites and accounts for 20% of cutaneous malignancies [16] having the overall risk of metastasis is in the range of 2-6%. In the case of lymph node metastasis, morbidity is meaningful;, altough 5-year survival rates as high as 73% have been achieved with the combination of surgical lymphadenectomy and radiation therapy [17]. Squamous cell carcinomas of head and neck region are locoregional, but pilar tumors are primarily and solely local. So they can be managed with wide local excision. Although the role of adjuvant radiation therapy in pilar tumor, especially in the malignant variant, is not very clear; adjuvant radiotherapy is justified considering the aggressive nature of the malignant variant and distant failures in previous series [18].

Dermoid cysts are rare subcutaneous cysts of ectodermal origin occur mostly on the face, forehead, neck, or scalp [19]. Clear-cell hydroadenocarcinoma [20] and cutaneous metastasis of renal cell carcinomas [18] may consist of the disease that we must be vigilant for the differential diagnosis of PTT.

In conclusion, our case of a 62 years old woman, having the history of trauma to her mass is mentioned about was macroscopically and microscopically benign. So, we have prefered follow-up and have not detect any recurrence during that period. We hope evaluation of well documented comprehensive series will let the improvement of more sophisticated prognostic schema for PTT s and it may asist in the solution of dilemma about malignant transformations of PTT and for the solution of differential diagnosis, also. It must been vigilant for the differential diagnosis and the rare malignant variant of this tumor even the metastasis from that malignant pilar tumor is extremely rare [20]. If these unfavorable probabilities are kept in view; as far as we are concerned, widely surgical excision with long-term surveillance may be the best choice for both diagnosis and treatment still today.

## Abbreviations

PTC: proliferating trichilemmal cyst; PTT: Proliferating trichilemmal tumour.

## Consent

Written informed consent was obtained from the patient for publication of this case report and accompanying images. A copy of the written consent is available for review by the Editor-in-Chief of this journal.

## Competing interests

The authors declare that they have no competing interests.

## Authors' contributions

DS provided the photos of the histopathological sections of the proliferating trichilemmal tumour and was the contributor in writing the manuscript, especially for the parts are related to histopathological assesments of the proliferating trichilemmal tumour and in the linguistic revision of the whole manuscript. IS performed the the preoperative, perioperative and the postoperative evaluation and the management of the patient, and was a major contributor in writing the manuscript. All authors read and approved the final manuscript.
